# Screening and Identification of *Trichoderma* Strains isolated from Natural Habitats in China with Potential Agricultural Applications

**DOI:** 10.1155/2021/7913950

**Published:** 2021-12-21

**Authors:** Ming Xue, Rui Wang, Chongyuan Zhang, Weiwei Wang, Fengtao Zhang, Di Chen, Sen Ren, Zhang Manman, Jumei Hou, Tong Liu

**Affiliations:** ^1^Key Laboratory of Green Prevention and Control of Tropical Diseases and Pests of Ministry of Education (College of Plant Protection, Hainan University), Haikou, Hainan 570228, China; ^2^Engineering Center of Agricultural Microbial Preparation Research and Development of Hainan (Hainan University), Haikou, Hainan 570228, China; ^3^Key Laboratory of Genetics and Germplasm Innovation of Tropical Special Forest Trees and Ornamental Plants of Ministry of Education (College of Forest, Hainan University), Haikou 570228, China

## Abstract

*Trichoderma* spp. are widely distributed in natural habitats and have been evaluated as a potential biocontrol agent (BCA) for disease control and plant growth promotion. In this study, 1308 *Trichoderma* strains were obtained from the plant rhizosphere soil, above-ground plants, and decaying wood from natural habitats in China. Among them, 49 *Trichoderma* strains showed a good inhibitory effect, especially against *Botrytis cinerea*, *Fusarium oxysporum*, and *Colletotrichum gloeosporioides* with inhibition rate above 85% in the dual culture test. Among these 49 strains, the 13 strains with broad-spectrum inhibitory effects also significantly promoted the seed germination of five crops (rice, cucumber, tomato, melon, and pakchoi) and root growth of four crop seedlings (watermelon, tomato, eggplant, and chili). Furthermore, these strains showed effective colonization in the rhizosphere and root of cucumber. *Trichoderma* strains SC012 and NX043 showed the highest chitinase and *β*-1,3-glucanase activity among all strains. Based on the morphological characterization and phylogenetic analysis of the nuclear ribosomal internal transcribed spacer (ITS) and translation elongation factor 1 (*tef1*), twelve *Trichoderma* strains were identified as *Trichoderma asperellum* and one as *Trichoderma afroharzianum*. This study suggests that the 13 *Trichoderma* strains are promising BCAs and could be developed as biofertilizers and biological pesticides for agricultural applications.

## 1. Introduction

Soil-borne diseases significantly decrease the quality and quantity of cash crops including vegetables, fruits, and officinal plants [1]. Most of the causal agents are polyphagous ubiquitous fungi, such as *F. oxysporum* causing wilt [2, 3], *C. gloeosporioides* causing anthracnose [4, 5], and *B. cinerea* causing gray mold [6, 7]. Currently available strategies for managing soil-borne pathogens include cultivation practices, physical control, and chemical control through resistant plants [8]. Although these approaches can reduce pathogen-caused losses, they are time-consuming and nontargeting and have poor control effects in some cases; chemical control is effective, but there are potential risks to the environment, and pathogens are prone to drug resistance [9]. Biological control is one of the best options to overcome the above-mentioned hurdles to the management of soil-borne plant pathogens and is undoubtedly more suitable for the sustainable development of modern agriculture [10].

*Trichoderma* spp. are recognized as important bioantagonists of phytopathogens in agricultural production and account for more than 60% of the registered biofungicides [11]. The biocontrol efficacy of *Trichoderma* is based on the activation of multiple mechanisms, including competing for nutrients and space, modifying the environmental conditions, and promoting plant growth and plant-defensive mechanisms, antibiosis, and mycoparasitism [12–15]. *T. asperellum* strain CCTCC-RW0014 exhibited antagonism activity to fusarium wilt disease due to its high hydrolytic activity of chitin, gelatin, carboxymethyl cellulose, and pachyman [16]. *T. virens* ZT05 showed a significant inhibitory effect on *R. solani*, and its mechanism of action was associated with hyperparasitism and antibiosis [17]; *T. harzianum* Ths97 can control *F. oxysporum* causing damping-off in *Pinus massoniana* seedlings by reducing reactive oxygen species, lowering lipid peroxidation and cell death, increasing osmolyte levels, stimulating the activities of antioxidant enzymes, and increasing soil fertility [18].

*Trichoderma* is one of the most diverse genera of the biocontrol fungi having above 300 reported species [19]. However, only a few of them are used in agricultural production as BCAs or biostimulating agents including *T. polysporum*, *T. hamatum*, *T. virens*, and *T. atroviride* [20–22]. Therefore, exploring the greater diversity of *Trichoderma* and evaluating its biocontrol effect have greater advantages in the biocontrol of plant pathogens. In addition, the success of BCAs is dependent upon the complex interactions between beneficial microbes with pathogens and plants [23]. It is a prerequisite for their effective practical application to understand how the biocontrol agents exert their protective effects [24]. In this study, we isolated 1308 *Trichoderma* strains various natural habitats in China and screened for their antifungal effect against a range of plant pathogenic fungi. Those strains that showed prominent antifungal activity were further tested for their plant growth and germination effect on a variety of important cash crops. The selected strains were then evaluated for root colonization ability, chitinase, and *β*-1,3-glucanase activity and finally identified on morphological and molecular basis.

## 2. Materials and Methods

### 2.1. Strains and Media

The pathogenic fungi (*B. cinerea* WN-2, *F. oxysporum* CS-1, *C. gloeosporioides* Penz QH-5, *Sclerotium rolfsii* SDF-23, *F. oxysporum f.* sp. *tracheiphilum* MTX-6, *Botryosphaeria dothidea* CTG-7, *Magnaporthe oryzae* DWM-3, *Botryodiplodia theobromae* PERF-1, *F. graminearum* KDR-1, *Rhizoctonia cerealis* ROD-18, *Stagonosporopsis cucurbitacearum* SD-3, and *R. solani* MNV-6) were isolated, identified, and stored at the Key Laboratory of Green Prevention and Control of Tropical Diseases and Pests of Hainan University for the subsequent studies.

*Trichoderma* selective medium (TSM) was used for the isolation of *Trichoderma* spp. [25]. Potato dextrose agar (PDA) medium was used to culture and preserve pathogenic fungi and *Trichoderma*. The *β*-1,3-glucanase synthetic medium (Yeast extract 30 g, NaNO_3_ 3 g, MgSO_4_·H_2_O 0.5 g, K_2_HPO_4_ 1 g, FeSO_4_·7H_2_O 0.01 g, KCl 0.5 g in 1 liter of distilled water, pH 6.0) and chitinase detection medium (chitin 5 g, MgSO_4_·7H_2_O 0.1 g, NH_4_NO_3_ 3 g, KH_2_PO_4_ 2 g in 1 liter of distilled water, pH 6.0) were used to ferment *Trichoderma* strains. Corn meal dextrose agar medium (CMD, 30 g corn flour, 18 g glucose, and 18 g agar; dilute the volume to 1 L with water) and synthetic low nutrient agar medium (SNA, 1.0 g KH_2_PO_4_, 10.5 g KCl, 1.0 g KNO3, 0.5 g MgSO4, 0.2 g glucose, 0.2 g sucrose, and 18 g agar; dilute it to 1 L with water) were used for the morphological identification of *Trichoderma* strains.

### 2.2. Collection of Samples and Isolation of *Trichoderma*

A total of 1018 samples of the rhizosphere soil and plants of cultivated crops from different ecological habitats, and decaying woods were collected from China. All samples were placed into sterile plastic bags in an icebox, transported to the laboratory in an icebox, and stored at 4°C for further use. Add 10 g of soil into 90 mL sterile water, shook for 30 min (200 r min^–1^), and then dilute to 10^3^-fold. Fresh plant samples and rotten woods were surface-disinfected by 70% alcohol for 30 s, ground in 1× PBS buffer, and diluted to 100-fold. The solutions (100 *μ*L) were added to the surface of TSM for the isolation of *Trichoderma* spp. Each sample was performed in triplicate. Morphologically different colonies were selected, transferred onto PDA medium, and purified by a single spore isolation [26]. All purified strains were maintained on PDA slants at 4°C for further use.

### 2.3. Inhibitory Effect of *Trichoderma* Strains on the Pathogenic Fungi

The antagonistic ability of all *Trichoderma* strains against pathogenic fungi was evaluated by a dual culture method. Five-millimeter diameter mycelial disks from *Trichoderma* strains and pathogenic fungi were transferred onto the border of PDA plate opposed to each other with a distance of 50 mm on Petri dishes (diameter, 90 mm) and incubated at 28°C for 7 days. The individual cultures of the pathogenic fungi were used as controls. The percentage of growth inhibition (PI) was calculated using the following equation: PI (%) = [(*C* − *T*)/*C*] × 100, where *C* represents radial growth of control mycelial and *T* represents radial growth of the treatment mycelial. Each treatment was performed in triplicate.

### 2.4. Effect of *Trichoderma* Strains on Seed Germination

Five crops (Supplementary Table S1), including rice, tomato, melon, cucumber, and pakchoi, were used to measure the effect of *Trichoderma* strains on seed germination. Uniform size 50 seeds of each crop were surface-disinfected by 75% ethanol for 1 min and 0.5% NaClO for 5 min and washed five times with sterile water. The seeds were soaked in Petri dishes containing 50 mL of *Trichoderma* strains spore suspension (10^5^ spores mL^–1^) for 2 h, transferred into new petri dishes covered with a layer of cotton and a filter paper, and cultured for 7 days at 28°C under the conditions of 12/12 h light/dark. The seeds treated with an equivalent volume of sterile water were used as control. The seed germination rate was calculated using the following equation: seed germination rate (%) = number of germinated seeds/number of total seeds tested × 100. The experiment was performed in triplicate.

### 2.5. Determination of Growth Promoting Effect of *Trichoderma* Strains

The root growth promotion experiment was performed at 28°C under the conditions of 12/12 h light/dark alternation with 80% relative humidity in a greenhouse. Uniform size seedlings of watermelon, chili, eggplant, and tomato were irrigated with a 100 mL spore suspension (10^5^ spores mL^–1^) of the *Trichoderma* strains. The seedlings treated with water were used as control. After 15 days, the seedlings were harvested, and the roots and aboveground seedlings were separated with scissors. The root length of seedlings was measured. The experiment was carried out in triplicate.

### 2.6. Determination of the Colonization Ability of *Trichoderma* Strains

The spore suspension (10^7^ spores mL^–1^) of the *Trichoderma* strains was added into sterilized composite soil containing vermiculite, matrix, and soil (1 : 3 : 7) and adjusted to a final concentration of 10^4^ spores g^–1^. Sterilized distilled water was used as the control. The *Trichoderma*-inoculated and *Trichoderma*-uninoculated soils were placed into sterile glass test tubes (height, 25 cm; inner diameter, 2 cm), and then, a cucumber seed was planted inside. All tubes were inserted with a cotton ball, sealed with tin foil, and placed in a plant growth chamber at 28°C under the conditions of 12/12 h light/dark alternation. After 15 days, the *Trichoderma* populations in the rhizosphere soil and roots endoderm of cucumber were calculated by a dilution plate method [27] (thirty seedlings for each treatment). The experiment was performed in triplicate.

### 2.7. Determination of *β*-1,3-Glucanase and Chitinase Activity of *Trichoderma* Strains

For the determination of *β*-1,3-glucanase activity, the *Trichoderma* strain was incubated in synthetic medium and cultured at 28°C with shaking at 180 rpm for 3 days. Medium without *Trichoderma* spp. was used as control. Approximately 1 mL of the culture filtrate was used to assay activity of *β*-1,3-glucanase using the 3,5-dinitrosalicylic acid colorimetric method [28]. For the determination of chitinase activity, *Trichoderma* strain was incubated in chitinase detection medium and cultured at 28°C with shaking at 180 rpm for 3 days. Colloidal chitin was prepared according to the method described by Roberts and Selitrennikoff [29]. The assay was performed in 10 mL tubes containing 1 mL 1% colloidal chitin, 1.5 mL 50 mM acetate buffer pH =5.0, and 1 mL suspension, the mixture was incubated at 40°C for 1 h, and the chitinase activity was determined basing the standard curves of N-acetyl-D-glucosamine (GlcNAc) measured at 540 nm absorbance. Enzyme bioactivity was calculated as follows: enzyme bioactivity (U/mL) = [(A1 − A2) + b1] × *d*/(K1 × V1 × T1), where A1 represents OD_540_ of samples, A2 represents OD_540_ of control, b1 represents the *y*-intercept of standard curves, *d* represents dilution rate of suspension, K1 represents the slope of standard curves, V1 represents the volume of mixture (mL), and T1 represents reaction time (h). Each treatment was performed in triplicate.

### 2.8. Identification of *Trichoderma* Strains

The morphological characteristics of conidia, phialide, conidiophore, and chlamydospores and the colony growth patterns of *Trichoderma* strains cultured on PDA, SNA, and CMD media were observed according to the description of Gams and Bissett [30]. For molecular identification, genomic DNAs of *Trichoderma* strains were extracted using the method of cetyl-trimethylammonium ammonium bromide (CTAB) as described by Ihrmark et al. [31]. The *tef-1* gene was amplified with primers *tef1*-728F (5′-CATCGAGAAGTTCGAGAAGG-3′) and *tef1-R* (5′-GCCATCCTTGGGAGATACCAGC-3′), and ITS region was amplified with primers ITS4 (5′-TCCTCCGCTTATTGATATGC-3′) and ITS5 (5′-GGAAGTAAAAGTCGTAACAAGG-3′). The PCR products were cloned into the Ti-19 vector and sequenced by the Sangon Biotechnology Co., Ltd. (Shanghai, China). All sequences from *Trichoderma* strains were aligned and spliced through “*tef-1*+ITS region” using MAFFT v7.215 [32]. The best substitution model (GTR+F+I+G4) was evaluated with IQ-TREE software using the ultrafast bootstrap method with 10,000 replicates. The phylogenetic tree was constructed based on *tef-1* gene and ITS region for the molecular identification of the *Trichoderma* species.

### 2.9. Statistical Analyses

All experiments were repeated three times. The data were analyzed using IBM SPSS Statistics 21.0 software (IBM Corp., Armonk, NY, USA) using one-way analysis of variance (ANOVA). Means of treatment were compared using the least significant difference (LSD) test at *P* < 0.05 and separated by superscript letters.

## 3. Results

### 3.1. Screening the Antagonistic Activity of *Trichoderma* Strains

A total of 1308 *Trichoderma* strains were obtained by the dilution plate method and single spore isolation method, including 1018 strains from 1018 rhizosphere soil samples, 205 strains from 45 rotten wood samples, and 86 strains from 16 plant samples. The dual culture test showed that 49 *Trichoderma* strains had good inhibitory effects against *F. oxysporum* (FOC), *C. gloeosporioides* (CG), and *B. cinerea* (BC), and their PIs were all exceeded 85% (Table 1). These 49 strains used for further experiments.

### 3.2. Promotion Effect of *Trichoderma* Strains on Seed Germination and Root Growth

The germination test showed that 13 strains (GZ070, HL100, HL119, HL135, HN059, JX013, SC012, XJ035, NX043, QH060, XJ087, SC098, and SC101) significantly promoted the seed germination of the five plants compared with the control. Among them, HL100 strain showed the best promotion effects on seed germination of rice, cucumber, and pakchoi, with increased germination rates of 12.17%, 13.23%, and 27.54%, respectively. The XM002-21 and XJ087 strains showed the highest promotion effects on seed germination of melon and tomato, with increased germination rates of 12.55% and 41.94%, respectively (Table 2).

The 13 *Trichoderma* strains that significantly promoted the seed germination were further tested for their effect on root growth of watermelon, chili, eggplant, and tomato seedlings. In greenhouse experiment, all strains showed a promotion effect on root growth of chili seedlings, and among them, HL100 strain showed maximum increase 12.17% in root length compared to control. Three strains (GZ070, HL100, and HN059) significantly promoted the root growth of watermelon seedlings. HN059 strain showed maximum increase of 18.81% in root length compared to control. *Trichoderma* strains GZ070, HL100, HN059, JX013, XJ087, and NX043 strains and HL119, HN059, SC012, XJ035, SC098, and SC101 were found to promote the root growth of eggplant and tomato seedlings, respectively. The highest increase in root length of eggplant (40.99%) and tomato plants (34.68%) was recorded by GZ070 and SC098 strains, respectively (Table 3).

### 3.3. Colonization Ability of *Trichoderma* Strains

**Figure 1:**
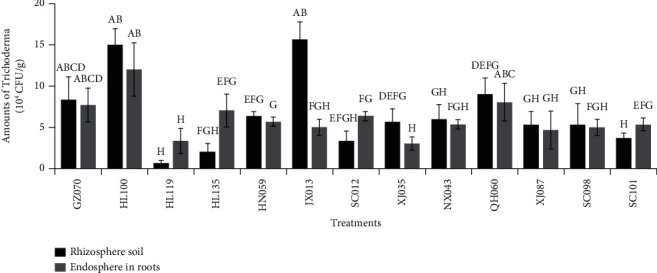
Determination of colonization ability of *Trichoderma* on cucumber seedlings. Data represent the mean ± SD (*n* = 3). Different letters indicate that the differences are significant (*P* < 0.05) using Duncan's multiple range test.

The colonization of GZ070, HL100, and JX013 strains in rhizosphere soil was higher than that of other strains, which were 8.30 × 10^4^, 1.50 × 10^5^, and 1.56 × 10^5^ CFU g^–1^, respectively. In the root endoderm of cucumber, the colonization of GZ070, HL100, and QH060 strains was higher than that of other strains, showing 7.67 × 10^4^, 1.20 × 10^5^, and 8.00 × 10^4^ CFU g^–1^, respectively (Figure 1).

### 3.4. Activity of Chitinase and *β*-1,3-Glucanase

The 13 *Trichoderma* strains were used to measure the activities of chitinase and *β*-1,3-glucanase. The results showed that these strains possessed a wide range of activity of chitinase and *β*-1,3-glucanase (Table 4). SC012 strain exhibited the highest chitinase activity (0.154 U mL^–1^). NX043 and XJ087 strains showed the highest *β*-1,3-glucanase activity. The lowest chitinase activity (0.041 U mL^–1^) and *β*-1,3-glucanase activity (0.1765 U mL^–1^) were shown by SC098 and XJ035 strains, respectively.

### 3.5. Detection of Antimicrobial Spectrum of *Trichoderma*

The results showed that 13 *Trichoderma* strains exhibited significant inhibitory effects on 9 kinds of pathogenic fungi (Table 5). The inhibition rates of 13 *Trichoderma* strains to FT and MO were all over 80% (80.81–88.37%). NX043 strain had the highest inhibition rate on FT (88.37%), and HL100 strain had the highest inhibition rate to MO (87.50%). However, the inhibition rate of 13 *Trichoderma* strains on BT was lower than that of other pathogenic fungi (59.49–70.26%).

### 3.6. Identification of *Trichoderma* Strains with Potential Agricultural Applications

**Figure 2:**
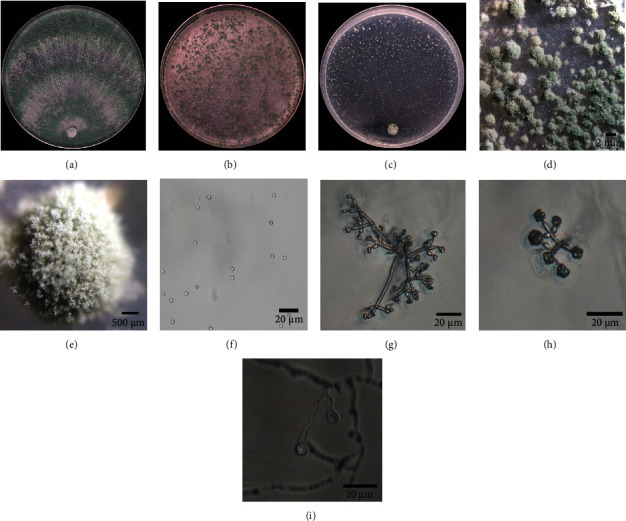
Morphological identification of JX013 (*Trichoderma asperellum*). (a–c) Cultures of JX013 that were grown on PDA, CMD, and SNA medium. (d, e) Conidial pustules. (f) Conidia. (g, h) Conidiophore. (i) Chlamydospores. PDA: potato dextrose agar medium; CMD: corn meal dextrose agar medium; SNA: synthetic low nutrient agar medium.

**Figure 3:**
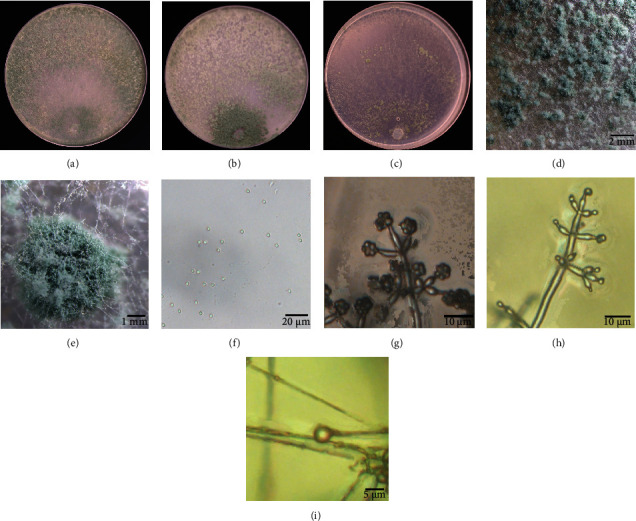
Morphological identification of SC098 (*Trichoderma afroharzianum*). (a–c) Cultures of JX013 which grew on PDA, CMD, and SNA medium. (d, e) Conidial pustules. (f) Conidia. (g, h) Conidiophore. (i) Chlamydospore. PDA: potato dextrose agar medium; CMD: corn meal dextrose agar medium; SNA: synthetic low nutrient agar medium.

**Figure 4:**
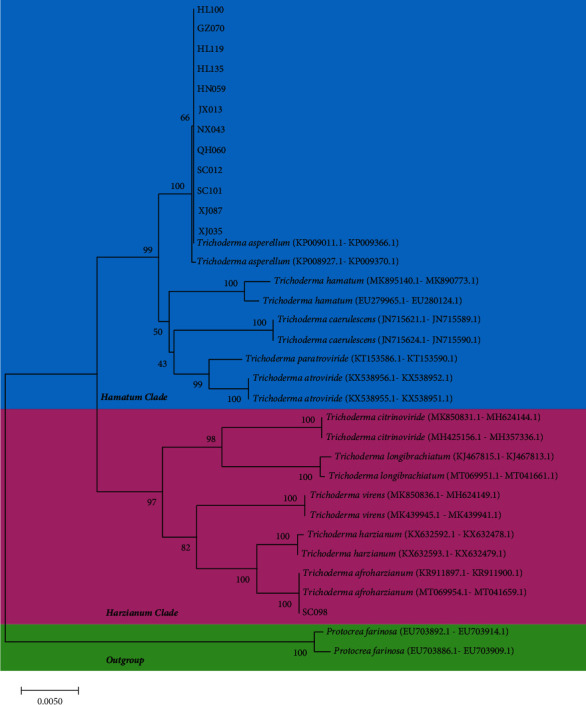
Phylogenetic analysis based on the *tef*-1 gene and ITS region from the strains screened. GenBank accession numbers of 13 *Trichoderma* strains shown in Supplementary Table 2.

All strains, except for SC098, showed the same morphological characteristics after growing on three kinds of medium for 7 days. These strains appeared as dark green colonies with a distinctive coconut odor on PDA plates, and they formed green conidial pustules on CMD medium and produced white, yellow, and green sorus, forming concentric zones on SNA plates (Figures 2(a)–2(e) and 3(a)–(e)). Conidia and chlamydospores are smooth, watered, and subglobose, and the branches were pyramidal and verticillate or paired lateral arising. Phialides were tended to cluster on branches. SC098 strain produced a diffuse yellow pigment on both sides of PDA plates, and green and yellow cottony sori were observed on CMD medium at 48 h. The mycelia of the strains were wavy and grown floccose to arachnoid on SNA plates, and phialides were ampulliform (length, 5-6.5 *μ*m; widest point, 2.9-3.2 *μ*m), tending to cluster and to create a narrow neck at the top (Figures 2(f)–2(h) and 3(f)–(h)). A phylogenetic tree demonstrated that all the selected strains had two distinct clades. JX013, HN059, XJ097, QH060, HL100, HL119, HL135, SC012, NX043, SC101, GZ070, and XJ035 strains belonged to the *Hamatum* clade and had affinity to KP009011 (*T. asperellum*). SC098 strain was identified as *T. afroharzianum*, which was closely related to KP0699541 (*T. afroharzianum*) and belonged to the *harzianum* clade (Figure 4). Based on the morphological and molecular characteristics, the remaining 12 strains were identified as *T. asperellum*, and SC098 strain was identified as *T. afroharzianum*.

## 4. Discussion

*Trichoderma* grows rapidly and can quickly occupy the growth space of pathogenic fungi, which is one of the important mechanisms of their antimicrobial effect [19]. The competitive effect of *Trichoderma* has been widely reported. For example, *T. asperellum* (T3, T4, T15, and T19) and *T. harzianum* T6 showed effective inhibition on graminicola in the dual culture assay [33]; *T. atroviride*, *T. harzianum*, and *T. viride* showed strong inhibitory effects on *Phytophthora*, *Botrytis cinerea*, and *Rhizoctonia solanacearum* in competitive bioassay tests [34]. In this study, we found that the growth rate of *Trichoderma* was significantly faster than that of pathogenic fungi. It could be clearly observed that *Trichoderma* covered most parts of the plates, and some can even continue to grow on the colony of pathogenic fungi and then covered the entire colony, showing obvious competition.

The production of cell wall-degrading enzymes (CWDEs) and volatile antibiotics is key parameters for *Trichoderma* as a biocontrol agent [35]. Saravanakumar et al. proved that there was a positive correlation between the CWDs of *Trichoderma* strains and its antagonism to pathogens [15]. We found that 13 *Trichoderma* strains (PI > 85%) with excellent antagonism to pathogens could secrete chitinase and *β*-1,3-glucanase, which were closely related to the cell wall composition of pathogens. Therefore, *Trichoderma* can not only compete with pathogens for space and nutrition but also degrade the cell walls of pathogens, deform, or even digest the hyphae, and inhibit the growth of pathogens. It has also been reported that the cell wall degrading enzymes such as chitinase and glucanase secreted by *Trichoderma* also play a vital role in the hyperparasitism process of *Trichoderma* on pathogens [36]. In addition, we also found that some *Trichoderma* strains had a distinctive coconut odor, which may be related to the production of the volatile compound namely 6-pentyl-2H-pyran-2-one (6PP) [37]. Wonglom et al. reported that *T. asperellum* T1 could produce 22 volatile compounds including 6PP, which could inhibit the growth of two leaf spot fungal pathogens, *Corynespora cassiicola* and *Curvularia aeria* [38].

This study confirmed that 13 *Trichoderma* strains had a good growth-promoting effect on many crops. *Trichoderma* promotes plant growth by enhancing nutrient absorption efficiency and the secretion of growth-promoting metabolites in host plant [39]. Previous studies have shown that *T. virens* promotes the growth of *Arabidopsis thaliana* by mediating the activation of the classical auxin response pathway [40]. Additionally, diverse VOCs from *T. asperellum* also contribute immensely to plant growth [37, 38]. *Trichoderma* T-22 could increase the efficiency of nitrogen application in maize, and under the same growth conditions, it could reduce the nitrogen application rate of corn by 40% [41]. Besides, *Trichoderma* can also induce plant hormone synthesis to promote plant growth by upregulating plant genes for hormone biosynthesis or downregulating hormone catabolism-related genes [42]. For instance, some reports suggested an indirect altered balance of the auxin hormone and cytokinin by *Trichoderma*, through the synthesis induction of these hormones by the plant, thereby achieving the purpose of promoting plant growth [43, 44].

As a mycorrhizal fungus, *Trichoderma* can successfully colonize in soil and plant roots, which is the premise of acting its biocontrol effect (including competition, growth promotion, antagonism, induced resistance) [45]. *Trichoderma* is precisely regulated in the process of colonizing plant roots. To successfully colonize roots, *Trichoderma* can instantly reduce the activity of root defense-system and promote its colonization in the root cortex [46]. Some reports suggested that root colonization by *Trichoderma* strains could increase levels of defense-related plant enzymes, including various peroxidases, chitinases, *β*-1,3-glucanases, and the lipoxygenase-hydroperoxide lyase pathway [47, 48]. In cucumber, root colonization by strain T-203 causes an increase in phenolic glucoside levels in leaves; their aglycones (which are phenolic glucosides with the carbohydrate moieties removed) are strongly inhibitory to a range of bacteria and fungi. Root colonization by these fungi therefore induces significant changes in the plant metabolic machinery [19]. In this study, we also evaluated the colonization ability of 13 *Trichoderma* strains on cucumber and found that all of them could colonize in the rhizosphere soil of cucumber, and the corresponding *Trichoderma* strain could also be isolated from other parts of cucumber, proving that they have strong colonization ability. However, the biocontrol effect of *Trichoderma* is the result of multiple mechanisms, and the interaction mechanism between *Trichoderma*, host plants, and pathogens needs to be further explored.

## 5. Conclusion

In this study, thirteen *Trichoderma* strains that showed high antagonistic potential against *B. cinerea*, *F. oxysporum*, and *C. gloeosporioides* were screened. At the same time, they could increase the germination rate of a variety of crop seeds, promote root growth, efficiently colonize in cucumber root soil, and show high *β*-1,3-glucanase and chitinase activities. These 13 strains of *Trichoderma* were identified as *T. asperellum* (12 strains) and *T. afroharzianum* (1 strain). These strains have good biological control potential, which provides a feasibility basis for the next evaluation of their field control effects and their application in production.

## Figures and Tables

**Table 1:** The region of the isolates which were screened in dual culture and its PI to three pathogens.

Code	Region	PI (%)		
		FOC	CG	BC
HL003	Hegang, Heilongjiang	85.13 ± 2.35	86.15 ± 1.54	90.56 ± 0.96
HN071	Ledong, Hainan	89.23 ± 1.54	88.72 ± 2.35	89.74 ± 1.18
SC006	Panzhihua, Sichuan	86.15 ± 6.71	97.95 ± 0.89	89.33 ± 6.11
SC017	Panzhihua, Sichuan	86.67 ± 2.35	93.85 ± 1.54	89.33 ± 2.31
HN059	Ledong, Hainan	85.13 ± 2.35	91.28 ± 0.89	88.89 ± 1.48
JX013	Pingxiang, Jiangxi	85.13 ± 3.20	88.21 ± 3.55	88.51 ± 5.27
HL007	Hegang, Heilongjiang	86.15 ± 1.54	91.28 ± 1.78	88.33 ± 0.22
HL101	Jiamusi, Heilongjiang	85.13 ± 0.89	88.21 ± 0.89	88.33 ± 3.89
HN046	Danzhou, Hainan	86.67 ± 0.89	89.74 ± 0.89	88.03 ± 1.48
HL008	Hailun, Heilongjiang	90.51 ± 0.44	95.49 ± 0.18	88.00 ± 0.92
JX002	Pingxiang, Jiangxi	85.64 ± 4.95	91.28 ± 0.89	87.03 ± 2.25
GX004	Guilin, Guangxi	88.21 ± 0.89	93.33 ± 0.89	87.03 ± 5.94
HL137	Hailun, Heilongjiang	86.15 ± 1.54	90.26 ± 3.20	87.03 ± 2.25
NM023	Chifeng, Neimeng	85.13 ± 2.35	91.28 ± 3.55	85.06 ± 1.99
HL119	Yichun, Heilongjiang	85.13 ± 3.87	85.13 ± 0.89	86.67 ± 2.31
FJ090	Ningde, Fujian	86.15 ± 1.54	95.90 ± 0.89	86.67 ± 2.31
HL092	Daqing, Heilongjiang	90.82 ± 0.09	93.33 ± 0.89	86.67 ± 2.31
ZJ019	Wenzhou, Zhejiang	88.72 ± 3.87	88.21 ± 3.20	86.50 ± 2.34
HN027	Danzhou, Hainan	85.13 ± 0.89	91.28 ± 0.89	86.32 ± 3.92
SX001	Changzhi, Shanxi	86.15 ± 1.54	88.72 ± 1.78	85.47 ± 1.48
HL144	Hegang, Heilongjiang	87.69 ± 1.54	92.82 ± 2.35	85.73 ± 4.49
HL018	Jiamusi, Heilongjiang	85.13 ± 0.89	91.79 ± 0.89	85.73 ± 5.94
HL132	Hegang, Heilongjiang	86.67 ± 4.70	89.74 ± 1.78	85.73 ± 5.94
HL131	Hegang, Heilongjiang	88.72 ± 0.89	91.28 ± 2.35	85.73 ± 5.94
FJ065	Ningde, Fujian	87.18 ± 3.87	91.28 ± 2.35	85.73 ± 4.49
SC011	Panzhihua, Sichuan	90.77 ± 3.08	93.85 ± 1.54	85.33 ± 2.31
SC018	Panzhihua, Sichuan	86.15 ± 1.54	96.41 ± 3.55	85.33 ± 2.31
SC013	Panzhihua, Sichuan	89.74 ± 3.20	91.79 ± 0.89	85.33 ± 6.11
FJ058	Ningde, Fujian	85.13 ± 0.89	94.36 ± 1.78	85.33 ± 2.31
HL015	Daqing, Heilongjiang	85.64 ± 0.89	91.79 ± 0.89	85.33 ± 2.31
HL100	Hailun, Heilongjiang	90.26 ± 3.20	95.90 ± 0.89	85.33 ± 4.62
FJ034	Fuzhou, Fujian	85.13 ± 1.78	85.64 ± 4.95	85.16 ± 4.67
LN005	Dalian, Liaoning	86.67 ± 1.78	87.18 ± 0.89	85.06 ± 3.98
XM002-21	Danzhou, Hainan	86.10 ± 0.09	86.15 ± 1.54	85.06 ± 5.27
HL048	Daqing, Heilongjiang	92.82 ± 0.89	96.41 ± 0.89	85.20 ± 2.08
SC010	Panzhihua, Sichuan	87.69 ± 2.66	90.77 ± 1.54	86.13 ± 3.70
HL135	Hegang, Heilongjiang	90.77 ± 1.54	90.26 ± 3.20	85.20 ± 2.12
SC012	Chengdu, Sichuan	90.21 ± 0.98	94.87 ± 3.20	85.33 ± 6.11
HN018	Danzhou, Hainan	90.26 ± 2.35	89.23 ± 1.54	85.21 ± 3.39
GZ070	Guiyang, Guizhou	90.15 ± 2.32	92.31 ± 1.54	86.50 ± 4.67
FJ087	Fuzhou, Fujian	88.72 ± 2.35	94.97 ± 0.71	85.33 ± 3.06
SC019	Panzhihua, Sichuan	89.74 ± 0.89	96.92 ± 2.66	85.33 ± 7.02
JS016	Xuzhou, Jiangsu	85.08 ± 0.80	89.74 ± 2.35	91.11 ± 1.92
BJ006	Haidian, Beijing	85.13 ± 2.35	86.67 ± 9.40	90.00 ± 3.33
QH060	Haixi, Qinghai	87.79 ± 0.18	87.69 ± 1.54	85.13 ± 2.35
SC101	Guangyuan, Sichuan	86.26 ± 0.18	85.13 ± 0.89	85.13 ± 0.89
NX043	Qinghai, Ningxia	85.13 ± 0.89	85.12 ± 0.89	89.23 ± 1.54
XJ035	Yili, Xinjiang	89.38 ± 0.27	85.13 ± 0.89	86.67 ± 1.78
SC098	Guangyuan, Sichuan	87.74 ± 0.09	85.17 ± 2.35	86.15 ± 0.27
XJ087	Kuerle, Xinjiang	86.15 ± 0.27	85.13 ± 0.89	85.13 ± 0.89

Data represent the mean ± SD (*n* = 3). FOC: *Fusarium oxysporum*; CG: *Colletotrichum gloeosporioides*; BC: *Botrytis cinerea*; PI (%): percentage of growth inhibition.

**Table 2:** The germination rate of 5 kinds of seeds after *Trichoderma* treatment.

Code	Seed germination rates (%)				
	Rice	Cucumber	Tomato	Melon	Pakchoi
GZ070	83.14 ± 1.97^a^	85.56 ± 10.18^a^	44.00 ± 14.37^d,e^	83.33 ± 10.00^a^	71.99 ± 4.41^b^
HL100	94.06 ± 3.98^a^	90.56 ± 7.97^a^	49.91 ± 15.25^b,c,d,e^	80.83 ± 0.72^a^	99.48 ± 0.90^a^
HL119	89.62 ± 4.36^a^	89.64 ± 7.50^a^	43.31 ± 1.30^d,e^	85.29 ± 7.50^a^	97.68 ± 0.86^a^
HL135	93.39 ± 3.26^a^	87.30 ± 10.13^a^	48.72 ± 15.06^c,d,e^	78.89 ± 8.67^a^	98.93 ± 0.93^a^
HN059	89.70 ± 3.14^a^	79.21 ± 7.40^a^	45.93 ± 8.41^d,e^	80.43 ± 3.25^a^	94.88 ± 2.46^a^
JX013	89.59 ± 7.09^a^	78.92 ± 13.81^a^	48.03 ± 5.85^c,d,e^	77.14 ± 8.51^a^	71.96 ± 5.24^b^
SC012	92.81 ± 3.92^a^	88.80 ± 4.74^a^	43.99 ± 18.12^d,e^	86.59 ± 11.48^a^	99.00 ± 1.72^a^
XJ035	83.78 ± 19.05^a^	82.22 ± 16.44^a^	72.00 ± 16.00^a,b,c^	76.81 ± 9.77^a^	78.67 ± 13.32^b^
NX043	82.33 ± 15.53^a^	80.00 ± 15.28^a^	74.67 ± 2.15^a,b^	82.22 ± 10.09^a^	81.11 ± 7.37^b^
QH060	87.00 ± 7.55^a^	85.56 ± 6.94^a^	88.00 ± 2.00^a^	77.78 ± 13.47^a^	83.33 ± 3.06^b^
XJ087	87.75 ± 8.49^a^	82.22 ± 5.09^a^	83.33 ± 5.77^a^	81.11 ± 1.92^a^	80.00 ± 3.46^b^
SC098	87.33 ± 7.02^a^	85.56 ± 8.39^a^	66.11 ± 10.46^a,b,c,d,e^	79.33 ± 4.62^a^	76.00 ± 10.58^b^
SC101	82.34 ± 12.17^a^	79.56 ± 1.92^a^	69.33 ± 1.15^a,b,c,d^	82.22 ± 11.71^a^	73.67 ± 11.72^b^
CK	81.91 ± 17.41^a^	77.33 ± 8.03^a^	41.39 ± 12.41^e^	76.34 ± 4.93^a^	71.94 ± 7.25^b^

Germination rates of seed were measured at 7 days, where seeds were treated by 10^5^ spore mL^–1^ suspension in 2 hours. “CK” means to treat seeds with sterile water. Data represent the mean ± SD (*n* = 3). Different letters indicate that the differences are significant (*P* < 0.05) using Duncan's multiple range test.

**Table 3:** Root length of different crops treated with a spore suspension at 15 days.

Code	Length of roots (cm)			
	Watermelon	Chili	Eggplant	Tomato
GZ070	11.71 ± 0.18^a,b,c^	9.56 ± 0.54^b,c,d^	12.88 ± 2.55^a^	3.74 ± 0.49^e,f,g,h^
HL100	12.26 ± 0.73^a,b,c^	11.85 ± 0.83^a,b^	10.48 ± 1.06^a,b,c,d,e^	3.60 ± 0.37^g,h^
HL119	9.36 ± 1.00^h^	11.08 ± 0.82^a,b,c^	9.47 ± 2.13^i,j^	5.03 ± 0.71^a,b,c,d^
HL135	11.20 ± 0.57^c,d,e,f,g^	9.72 ± 0.74^b,c,d^	9.54 ± 1.29^h,i,j^	2.98 ± 0.16^h^
HN059	13.03 ± 1.51^a^	11.28 ± 1.38^a,b^	10.16 ± 0.55^c,d,e,f,g^	4.10 ± 0.29^e,f,g,h^
JX013	9.71 ± 0.54^h^	10.33 ± 0.57^a,b,c,d^	10.91 ± 1.02^a,b,c,d^	3.73 ± 0.46^f,g,h^
SC012	11.33 ± 1.10^b,c,d,e,f^	10.19 ± 0.95^a,b,c,d^	9.24 ± 0.66^h,i,j^	5.02 ± 0.54^a,b^
XJ035	10.69 ± 0.49^c,d,e,f,g^	10.96 ± 0.90^a,b,c,d^	8.24 ± 0.70^h,i,j^	4.76 ± 0.54^b,c,d^
NX043	11.65 ± 1.66^b,c,d,e,f,g^	10.51 ± 0.68^a,b,c,d^	9.39 ± 1.58^e,f,g,h,i,j^	3.37 ± 0.38^h^
QH060	11.50 ± 0.99^c,d,e,f,g^	9.20 ± 0.51^a,b,c,d^	8.45 ± 0.57^h,i,j^	3.43 ± 0.61^f,g,h^
XJ087	10.25 ± 0.58^g,h^	9.79 ± 0.78^a,b,c,d^	11.33 ± 0.38^a,b,c,d,e^	3.35 ± 0.33^f,g,h^
SC098	10.18 ± 0.88^e,f,g,h^	8.96 ± 0.76^b,c,d^	8.45 ± 0.87^i,j^	5.40 ± 0.46^a^
SC101	9.99 ± 0.83^f,g,h^	9.88 ± 0.65^a,b^	7.00 ± 0.32^k^	4.42 ± 0.37^d,e,g,f^
CK	11.26 ± 0.18^a,b^	9.34 ± 0.38^d^	9.63 ± 0.15^f,g,h,i,j^	3.93 ± 0.14^f,g,h^

The concentration of the conidial suspension was 10^5^ spores mL^–1^. “CK” means to treat seeds with sterile water. Data represent the mean ± SD (*n* = 3). Different letters indicate that the differences are significant (*P* < 0.05) using Duncan's multiple range test.

**Table 4:** Chitinase and *β*-1,3-glucanase activity of *Trichoderma* isolates at 3 days in enzyme synthetic medium.

Code	Chitinase (U mL^–1^)	*β*-1,3-Glucanase (U mL^–1^)
GZ070	0.069 ± 0.018^g,h,i^	0.398 ± 0.044^b,c^
HL100	0.105 ± 0.017^b,c,d,e,f^	0.319 ± 0.042^b,c,d^
HL119	0.112 ± 0.018^b,c,d,e^	0.397 ± 0.022^b,c^
HL135	0.081 ± 0.006^e,f,g,h^	0.488 ± 0002^b^
HN059	0.068 ± 0.016^g,h,i^	0.340 ± 0.002^b,c,d^
JX013	0.082 ± 0.001^e,f,g,h^	0.489 ± 0.05^b^
SC012	0.154 ± 0.013^a^	0.3744 ± 0.062^b,c,d^
XJ035	0.087 ± 0.009^d,e,f,g,h^	0.1765 ± 0.038^c,d^
NX043	0.108 ± 0.028^b,c,d,e^	0.8162 ± 0.172^a^
QH060	0.126 ± 0.009^b^	0.2258 ± 0.070^b,c,d^
XJ087	0.104 ± 0.039^b,c,d,e,f^	0.7993 ± 0.199^a^
SC098	0.047 ± 0.015^i^	0.3814 ± 0.048^b,c,d^
SC101	0.095 ± 0.006^c,d,e,f,g^	0.3170 ± 0.08^b,c,d^

Data represent the mean ± SD (*n* = 3). Different letters indicate that the differences are significant (*P* < 0.05) using Duncan's multiple range test.

**Table 5:** Inhibition spectrum of *Trichoderma* isolates, the PI of isolates against 9 pathogens in dual culture.

Code	PI (%)								
	SR	FT	BD	MO	BT	FG	RC	SC	RS
GZ070	78.97 ± 1.78^a^	87.50 ± 1.33^a^	83.61 ± 4.09^a,b^	83.62 ± 1.49^a^	67.69 ± 1.54^a^	83.08 ± 1.54^a^	83.97 ± 1.11^c^	78.09 ± 1.65^a,b^	85.29 ± 3.23^a,b^
HL100	75.38 ± 4.07^b^	87.50 ± 2.80^a^	86.34 ± 2.3^7a,b^	87.50 ± 1.98^a^	62.50 ± 2.35^a^	81.03 ± 0.89^a,b^	85.58 ± 0.96^b,c^	79.17 ± 0.62^a,b^	76.31 ± 1.41^b^
HL119	79.49 ± 0.89^a^	88.08 ± 0.50^a^	90.44 ± 4.73^a^	86.64 ± 0.75^a^	68.72 ± 0.89^a^	82.05 ± 0.89^a,b^	85.90 ± 1.11^b,c^	79.17 ± 2.49^a,b^	85.70 ± 4.64^a^
HL135	78.46 ± 2.66^a^	86.34 ± 0.50^c^	78.14 ± 4.73^b,c^	84.05 ± 1.98^a^	65.64 ± 1.78^a^	84.36 ± 1.18^a^	85.26 ± 1.11^b,c^	78.09 ± 0.62^a,b^	84.89 ± 3.08^a,b^
HN059	78.56 ± 0.18^a^	86.63 ± 0.50^a^	87.70 ± 4.10^a,b^	86.21 ± 3.73^a^	65.64 ± 9.01^a^	81.28 ± 1.94^a,b^	81.41 ± 1.11^b,c^	78.45 ± 2.85^a,b^	82.84 ± 4.42^a,b^
JX013	80.00 ± 5.33^a^	86.45 ± 1.31^a^	81.56 ± 2.05^a,b,c^	85.78 ± 2.59^a^	64.10 ± 0.89^a^	83.85 ± 2.77^a^	83.33 ± 1.06^c^	82.04 ± 5.42^a,b^	85.70 ± 3.54^a^
SC012	78.97 ± 3.55^a^	86.05 ± 0.87^a^	84.29 ± 1.18^a,b^	86.64 ± 1.49^a^	70.26 ± 8.75^a^	82.15 ± 2.51^a,b^	87.82 ± 1.11^a,b^	79.89 ± 3.29^a,b^	83.66 ± 3.54^a,b^
XJ035	76.92 ± 2.66^a^	86.92 ± 0.87^a^	86.34 ± 4.27^a,b^	84.91 ± 0.75^a^	69.23 ± 7.99^a^	82.05 ± 0.44^a,b^	83.97 ± 1.11^c^	81.68 ± 5.39^a,b^	88.15 ± 3.54^a^
NX043	79.74 ± 0.44^a^	88.37 ± 1.01^a^	82.24 ± 4.27^a,b,c^	86.64 ± 0.75^a^	61.03 ± 1.78^a^	83.13 ± 0.09^a^	89.42 ± 0.96^a^	76.65 ± 0.62^a,b^	86.11 ± 3.94^a^
QH060	79.49 ± 0.89^a^	80.81 ± 1.74^b,c^	80.19 ± 1.18^a,b,c^	85.78 ± 2.24^a^	64.62 ± 11.99^a^	83.08 ± 1.54^a^	84.94 ± 1.47^b,c^	75.57 ± 1.24^b^	81.21 ± 2.83^a,b^
XJ087	78.46 ± 1.54^a^	84.88 ± 1.82^b^	81.56 ± 2.05^a,b,c^	84.05 ± 1.49^a^	69.23 ± 6.15^a^	82.05 ± 1.78^a,b^	83.97 ± 1.11^c^	78.45 ± 3.73^a,b^	87.75 ± 2.45^a^
SC098	76.92 ± 4.07^a^	88.08 ± 1.33^a^	72.68 ± 6.26^c^	82.76 ± 1.49^a^	66.67 ± 6.22^a^	78.97 ± 0.89^b^	79.49 ± 2.94^d^	73.42 ± 1.24^a,b^	87.13 ± 0.87^a^
SC101	76.41 ± 5.40^b^	84.59 ± 3.30^b^	89.07 ± 6.26^a^	83.19 ± 1.29^a^	59.49 ± 4.44^a^	82.31 ± 1.33^a,b^	84.29 ± 1.47^c^	85.27 ± 5.53^a^	81.62 ± 6.13^a,b^

Data represent the mean ± SD (*n* = 3). Different letters indicate that the differences are significant (*P* < 0.05) using Duncan's multiple range test. SR: *Sclerotium rolfsii*; FT: *Fusarium oxysporum* f.sp. tracheiphilum; BD: *Botryosphaeria dothidea*; MO: *Magnaporthe oryzae*; BT: *B. theobromae*; FG: *F. graminearum*; RC: *Rhizoctonia cerealis*; SC: *Stagonosporopsis cucurbitacearum*; RS: *R. solani*; PI: percentage of growth inhibition.

## Data Availability

Data are available on request.

## Supplementary Materials

Supplementary Materials Supplementary Table 1 shows the types, cultivar, and sources of plant seeds or seedlings used in the study. Supplementary Table 2 shows the GenBank accession numbers of 13 *Trichoderma* strains.
