# Supplementary material for: Screening and Identification of Trichoderma Strains isolated from Natural Habitats in China with Potential Agricultural Applications
Source: Biomed Res Int. 2021 Dec 21;2021:7913950. doi: 10.1155/2021/7913950 (PMC8714372; doi:10.1155/2021/7913950)
Supplement: Supplementary Materials — Supplementary Table 1 shows the types, cultivar, and sources of plant seeds or seedlings used in the study. Supplementary Table 2 shows the GenBank accession numbers of 13 Trichoderma strains. [file 7913950.f1.docx]

Supplemental Table

Table S1. The seeds scource use for seeds germination, colonization assay and plant promotion in these study

| Seeds | Cultivar | Provider |
| --- | --- | --- |
| Rice (*Oryza sativa* L.) | Zhongyou 233 | Tropical Crop Germplasm Research Institute (Wenchang, China) |
| Tomato (*Solanum lycopersicm*) | Xinfan 2 | Xinjiang Academy of Agricultural Sciences (Xinjiang, China ) |
| Melon (*Cucumis melo* L.) | Maohuayangjiaocui | Zhengzhou Fruit research Institute (Zhengzhou, China) |
| Cucumber (*Cucumis sativus* L.) | Zhongnong 8 | Institute of Vegetables and Flowers, Chinese Academy of Agricultural Science (Beijing, China) |
| Pakchoi (*Brassica chinensis* L.) | K008 | Xingning Qingfeng Yingke seed Co., Ltd (Xingning, China) |
| Watermelon (*Citrullus lanatus* (Thunb.) Matsum. et Nakai) | Jingxin 1 | Hanyu seed Co., Ltd (Hebei, China) |
| Chili (*Capsicum annuum* L.) | Huayu 8898 | Qingxian Xingyun Vegetable Breeding Center (Cangzhou, China） |
| Eggplant (*Solanum melongena* L.) | Jingqie 10 | Beijing acamdemy of Agriculture and Forestry Science (Beijing，China) |

Table S2. GenBank accession numbers of 13 *Trichoderma* strains

| Strains | GenBank accession numbers (*tef-1*) | GenBank accession numbers (ITS) |
| --- | --- | --- |
| GZ070 | OK338646 | OK335772 |
| HL100 | OK338645 | OK335771 |
| HL119 | MN905715 | OK335770 |
| HL135 | MN905716 | OK335769 |
| HN059 | OK338644 | OK335768 |
| JX013 | MN905717 | OK335767 |
| NX043 | OK338643 | OK335766 |
| QH060 | OK338642 | OK335765 |
| SC012 | MN905718 | OK335764 |
| SC098 | OK338641 | OK335763 |
| SC101 | OK338640 | OK335762 |
| XJ035 | MN905719 | OK335760 |
| XJ087 | OK338639 | OK335761 |
